# Rapid Epidemic Expansion of Chikungunya Virus East/Central/South African Lineage, Paraguay

**DOI:** 10.3201/eid2909.230523

**Published:** 2023-09

**Authors:** Marta Giovanetti, Cynthia Vazquez, Mauricio Lima, Emerson Castro, Analia Rojas, Andrea Gomez de la Fuente, Carolina Aquino, Cesar Cantero, Fatima Fleitas, Juan Torales, Julio Barrios, Maria J. Ortega, Maria L. Gamarra, Shirley Villalba, Tania Alfonzo, Joilson Xavier, Talita Adelino, Hegger Fritsch, Felipe C.M. Iani, Glauco C. Pereira, Carla de Oliveira, Gabriel Schuab, Evandra S. Rodrigues, Simone Kashima, Juliana Leite, Lionel Gresh, Leticia Franco, Houriiyah Tegally, Wesley C. Van Voorhis, Richard Lessels, Ana Maria Bispo de Filippis, Andrea Ojeda, Guillermo Sequera, Romeo Montoya, Edward C. Holmes, Tulio de Oliveira, Jairo M. Rico, José Lourenço, Vagner Fonseca, Luiz C.J. Alcantara

**Affiliations:** Università Campus Bio-Medico di Roma, Italy (M. Giovanetti);; Instituto Oswaldo Cruz, Belo Horizonte, Brazil (M. Giovanetti, M. Lima, E. Castro, J. Xavier, H. Fritsch, L.C.J. Alcantara);; Laboratorio Central de Salud Pública, Asunción, Paraguay (C. Vazquez, A. Rojas, A. Gomez de la Fuente, C. Aquino, C. Cantero, F. Fleitas, J. Torales, J. Barrios, M.J. Ortega, M.L. Gamarra, S. Villalba, T. Alfonzo);; Central de Saúde Pública do Estado de Minas Gerais, Ezequiel Dias, Brazil (M. Lima, E. Castro, T. Adelino, F.C.M. Iani, G.C. Pereira);; Universidade Federal de Minas Gerais, Belo Horizonte (J. Xavier, H. Fritsch);; Instituto Oswaldo Cruz, Rio de Janeiro, Brazil (C. de Oliveira, G. Schuab, A.M.B. de Filippis);; University of São Paulo, São Paulo, Brazil (E.S. Rodrigues, S. Kashima);; Pan American Health Organization/World Health Organization, Washington, DC, USA (J. Leite, L. Gresh, L. Franco, J.M. Rico);; Stellenbosch University, Stellenbosch, South Africa (H. Tegally, T. de Oliveira);; University of KwaZulu-Natal, Durban, South Africa (H. Tegally, R. Lessels, T. de Oliveira);; National Institutes of Health, Bethesda, Maryland, USA (W.C. Van Voorhis);; Dirección General de Vigilancia de la Salud, Asunción (A. Ojeda, G. Sequera);; Organización Panamericana de la Salud/Organización Mundial de la Salud Asuncion (R. Montoya);; University of Sydney, Sydney, New South Wales, Australia (E.C. Holmes);; University of Lisbon, Lisbon, Portugal (J. Lourenço);; Organização Pan-Americana da Saúde/Organização/Mundial da Saúde, Brasilia, Brazil. (V. Fonseca)

**Keywords:** chikungunya virus, viruses, rapid epidemic expansion, East/Central/South African lineage, ECSA lineage, genomic monitoring, Paraguay, vector-borne infections, meningitis/encephalitis

## Abstract

The spread of Chikungunya virus is a major public health concern in the Americas. There were >120,000 cases and 51 deaths in 2023, of which 46 occurred in Paraguay. Using a suite of genomic, phylodynamic, and epidemiologic techniques, we characterized the ongoing large chikungunya epidemic in Paraguay.

Chikungunya is a mosquitoborne disease caused by the chikungunya virus (CHIKV), a single-stranded positive-sense RNA virus belonging to the family *Togaviridae* (*1*), which is transmitted to humans through the bite of infected *Aedes aegypti* and *Ae. albopictus* mosquitoes. This disease is generally an acute, self-limiting illness characterized by fever and severe joint pain, although persistent or relapsing joint pain can occur (*1*). Atypical and severe manifestations (including meningoencephalitis) have been reported, and death is usually associated with older ages and other underlying diseases. Mother-to-child transmission of CHIKV occurs, and neonatal disease can be severe, with neurologic, myocardial, or hemorrhagic complications (*1*).

CHIKV can be classified into 4 distinct genotypes: West African, East/Central/South African (ECSA), Asian, and Indian Ocean lineages (*2*,*3*). An imported case of CHIKV in Paraguay was detected in June 2014 in a person from the Dominican Republic (*4*). Using on-site genomic monitoring, phylodynamic and epidemiologic approaches, we characterized the large-scale and ongoing CHIKV epidemic in Paraguay.

##  The Study

This study was reviewed and approved by the Pan American Health Organization (PAHO) Ethics Review Committee (PAHO no. 2016-08-0029) and by the Paraguayan Ministry of Public Health and Social Welfare (MSPyBS/SG no. 0944/18). Samples used in this study were deidentified residual samples from routine diagnosis of arboviruses in the Paraguayan Public Health Laboratory, which is part of the public network within the Paraguayan Ministry of Health.

We partnered with PAHO to perform on-site genomic surveillance at the Laboratorio Central de Salud Pública in Asunción, Paraguay. During March 11‒17, 2023, a team of molecular biologists from Brazil and Paraguay worked with selected samples (based on cycle threshold [Ct] values <35 and availability of epidemiologic metadata, generating 179 viral genomes deposited in GenBank under accession nos. OQ775394‒567 and OQ567722‒5). We performed sequencing by using Nanopore technology (*5*). We constructed phylogenetic trees to explore the evolutionary and epidemiologic relationships of CHIKV in Paraguay with those of other sequences of this viral genotype sampled globally. We retrieved from GenBank 715 CHIKV ECSA genome sequences collected through March 30, 2023, with associated lineage date and country of collection,. We compiled a description of the relevant methods used (Appendix 1) and strains analyzed (Appendix 2).

**Figure 1:**
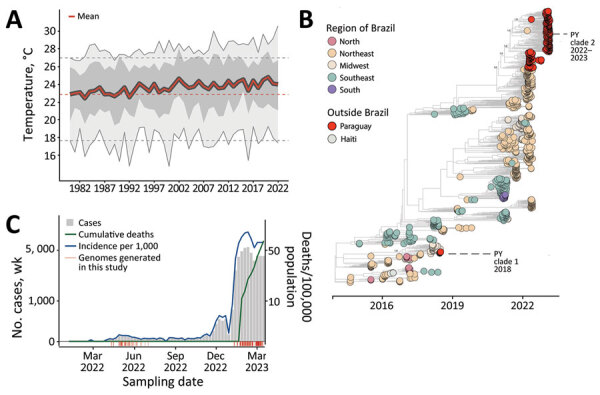
Spatial and temporal distribution of cases of chikungunya in Paraguay. A) Temperature trends during 1981‒2022. Yearly mean (red line), yearly minimum and maximum (light gray shading), yearly 50% quantiles (dark gray shading), minimum and maximum temperatures in 1981 (dashed gray lines) and mean temperature in 1981 (dashed red line) are shown. B) Number of chikungunya virus genome sequences in Paraguay compared with Brazil (by region) and Haiti. Size of circles indicates number of new genomes generated in this study. C) Weekly reported chikungunya cases (gray area), incidence normalized per 100,000 persons (blue line), and cumulative deaths (green line -or other color if you need to change this line color) during 2022–2023 (through epidemiologic week 11). Red bars indicate dates of sample collection of genomes generated in this study.

Autochthonous infections were detected in Paraguay in 2015, and CHIKV has been detected in the country every year since that date (Appendix 1 Figure 1, panel A). On the basis of reported suspected CHIKV infections, Paraguay has had 4 epidemic waves, in 2015, 2016, 2018, and 2023, all associated with summer months (Appendix 1 Figure 1, panel A). During October 2, 2022‒April 10, 2023, a total of 118,179 suspected and confirmed infections were reported, including 3,510 hospitalized case-patients and 46 deaths (*4*,*6*). Neonates have accounted for 0.3% (n = 162) of these cases and 8 deaths. In addition, 294 suspected cases of acute meningoencephalitis have been reported, 125 (43%) of which have been attributed to CHIKV (*5*,*6*).

Although yearly minimum temperatures across Paraguay have remained stable over the past 40 years, mean and maximum yearly temperatures have been steadily increasing, and the rapid and large resurgence of CHIKV in 2022 coincided with the highest mean temperatures reported (Figure 1, panel A). Before 2022, confirmed infections were restricted to the Central, Paraguarí, and Amambay Districts; the Central District dominated the reports (Appendix 1 Figure 1, panel B). After viral resurgence in 2022, confirmed infections have been reported in all districts (Appendix 1).

**Figure 2:**
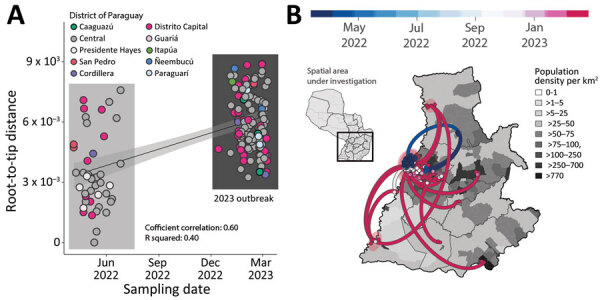
Expansion of the chikungunya East/Central/South/African lineage epidemic in Paraguay. A) Regression of root-to-tip genetic distances and sampling dates estimated by using TempEst version 1.5.3, (http://tree.bio.ed.ac.uk/software/tempest), buffers (shaded area) representing 90% CIs. Colors indicate geographic location of sampling. B) Spatiotemporal reconstruction of the spread of CHIKV ECSA in Paraguay. Circles represent nodes of the maximum clade credibility phylogeny, colored according to their inferred time of occurrence (scale shown). Shaded areas represent 80% highest posterior density interval and depict uncertainty of the phylogeographic estimates for each node. Solid curved lines indicate links between nodes and directionality of movement. Differences in population density are shown on a gray-white scale.

We screened 179 quantitative reverse transcription PCR‒positive samples for CHIKV. All contained sufficient DNA (>2 ng/μL) to proceed with library preparation, and their PCR Ct values were a mean of 21 (range 9‒34) (Appendix 2). Samples had good spatial representation of southern Paraguay (10/17 districts) (Figure 1, panel B), including several districts that had the highest historical counts of CHIKV infections (Appendix 1) and captured the out-season and in-season periods of transmission (autumn and early winter 2022 and summer 2023) (Figure 1, panel C). Analysis of sample sequence coverage versus Ct showed an average coverage of 94% among samples and a Ct of 28, below which average coverage >90% was achieved (Figure 2; Appendix 1). Most genomes (87%) were obtained from serum samples, the rest from cerebrospinal fluid; 54% were from female and 46% from male patients, and the mean age of the samples was 41 (range 26‒95) days (Appendix 2).

Most (58%) genomes were from CHIKV infection outcomes in outpatients, followed by fatal (18%), intensive care unit (17%) and inpatient (7%) infections (Figure 1; Appendix 1). Compared with outpatient outcomes, we found a clear association of fatal outcomes in older age groups (Figure 1). The same comparison with outcomes requiring medical attention (ICU, inpatients) was not statistically significant (Figure 1). This observation contrasted the common notion that CHIKV symptomatic infections are more frequent in older age groups (*7*).

To determine the dynamics of the CHIKV ECSA in Paraguay, we performed phylodynamic analysis of a dataset comprising 715 available representative genomes combined with viruses sequenced in this study (n = 179, collected during April 6, 2022‒March 10, 2023) (Figure 1). A date-stamped phylogeny indicated that all the novel isolates formed a single, large, well-supported monophyletic group, denoted Paraguay clade 2, within the CHIKV ECSA American clade. This result strongly suggests that the 2022–2023 epidemic was not related to cross-border transmission from Brazil, as reported (*8*) (Figure 1), but was more likely the result of continual transmission within Paraguay over a period of 11 months of a viral strain that was introduced in the region in early 2022 (Figures 1, 2).

To investigate evolution of the Paraguay clade 2 in more detail, we used a smaller dataset (n = 179) representing this virus clade in isolation. We found a relatively strong correlation between sampling date and root-to-tip genetic divergence in this dataset (r^2^ = 0.40, correlation coefficient = 0.60), indicating relatively clock-like virus evolution (Figure 2). Phylogeographic analysis of Paraguay clade 2 enabled reconstruction of viral movements among different districts in Paraguay (Figure 2) and suggested a mean time of origin in late March 2022 (95% highest posterior density March 25, 2022‒April 5, 2022). Viruses from this clade spread multiple times from the Midwestern District (Distrito Capital and Central Regions) toward the Southeast (Itapúa) and to the Midwest, as indicated by virus sequences from the Presidente Hayes and the Cordillera Regions (Figure 2).

Virus transmission dynamics roughly followed patterns of population density, moving most often between the most populous urban localities (Figure 1 panel B; Figure 2). Because it is recognized that both nonsynonymous and synonymous mutations can lead to changes in viral RNA (*9*,*10*), affecting splicing, stability, translation, or cotranslational protein folding, additional studies will be necessary to determine the potential effects of mutations on structure and function and, thus, on viral pathogenesis and fitness.

## Conclusions

This study highlights the resurgence of CHIKV ECSA in Paraguay during 2022–2023. Our findings provide evidence of lineage persistence over a period of 11 months preceding resurgence and report the notable coincidence of virus resurgence and the highest mean temperatures recorded in Paraguay. Those 2 factors, combined with presence of the vectors and a large proportion of the population susceptible to CHIKV probably generated an ideal scenario for the observed fast and large CHIKV epidemic wave that started at the end of 2022. Given the association of ongoing resurgence with a specific lineage of CHIKV ECSA with 2 synonymous changes in nonstructural proteins 3 and 4 and uncertainty of how the ongoing epidemic will unfold, genomic surveillance should remain active to track real-time evolution and spatial spread, contributing to public health risk assessments in Paraguay and other countries in South America.

Appendix 1 Additional information on rapid epidemic expansion of chikungunya virus East/Central/South African lineage, Paraguay.

Appendix 2 Virus strains used in study of rapid epidemic expansion of chikungunya virus East/Central/South African lineage, Paraguay.
