# Supplementary material for: Rapid Epidemic Expansion of Chikungunya Virus East/Central/South African Lineage, Paraguay
Source: Emerg Infect Dis. 2023 Sep;29(9):1859–63. doi: 10.3201/eid2909.230523 (PMC10461647; doi:10.3201/eid2909.230523)
Supplement: Appendix 1 — Additional information on rapid epidemic expansion of chikungunya virus East/Central/South African lineage, Paraguay. [file 23-0523-Techapp-s1.pdf]

Article DOI: https://doi.org/10.3201/eid2909.230523

EID cannot ensure accessibility for Supplemental Materials supplied by authors. Readers who have difficulty accessing supplementary content should contact the authors for assistance.

Rapid Epidemic Expansion of Chikungunya Virus East/Central/South African Lineage, Paraguay

Appendix 1

Methods and Data

Sample Collection and Whole-Genome Sequencing

A total of 179 samples (n = 156 serum, and n = 23 cerebrospinal fluid [CSF]) retrieved from patients presenting symptoms compatible with arboviral infection were collected by the Laboratorio Central de Salud Pública of Paraguay, in Asunción for molecular diagnosis. Samples were submitted first to nucleic acid extraction using the QIAmp Viral RNA Mini Kit (QIAGEN) and then subjected to real-time reverse transcription PCR (RT-qPCR) for arbovirus detection (including ZIKV, CHIKV, ad DENV 1–4) (*1–3*). Genome sequencing was conducted using the Nanopore technology (*4*). Briefly, viral RNA was submitted to first-strand cDNA synthesis. Then, a multiplex tiling PCR was conducted using Q5 High Fidelity Hot-Start DNA Polymerase (New England Biolabs) and CHIKV sequencing primers (*4*). DNA library preparation was conducted by using the Ligation Sequencing Kit and the Native Barcoding Kit (NBD104, Oxford Nanopore Technologies, Oxford, UK) (*4*). The final normalized sequencing library was loaded onto a R9.4 flow cell, and data were collected for 6 hours. FAST5 files were base called using Guppy and demultiplexing was performed using guppy software. Consensus sequences were obtained by hybrid assembly approach using Genome Detective (https://www.genomedetective.com/) (*5*). A total of 40,177 mapped reads were obtained, resulting in a sequencing mean depth >1,000X and a coverage of >94%, confirming CHIKV-ECSA genotype (Appendix 2).

Sequences were aligned using MAFFT (*6*) and edited using AliView (*7*). These datasets were assessed for the presence of phylogenetic signals by applying the likelihood mapping analysis implemented in the IQ-TREE2 software (*8*). A maximum likelihood phylogeny was reconstructed using IQ-TREE2 software under the HKY + G4 substitution model (*8*). We inferred time-scaled trees using TreeTime (*8*). The presence of a temporal signal was evaluated in TempEst (*9*), and time-scaled phylogenetic trees were inferred using the BEAST package (*10*). We used a stringent model selection analysis with path-sampling (PS) and steppingstone (SS) procedures to estimate the most appropriate molecular clock model for the Bayesian phylogenetic analysis (*11*). The uncorrelated relaxed molecular clock model was chosen for all datasets as indicated by estimating marginal likelihoods, also using the codon based SRD06 model of nucleotide substitution and the nonparametric Bayesian Skyline coalescent model. To model the phylogenetic diffusion of detected 2022–2023 transmission clade we used a flexible relaxed random walk diffusion model (*12*,*13*) that accommodates branch-specific variation in rates of dispersal with a Cauchy distribution and a jitter window site of 0.01 (*14*,*15*). For each sequence, coordinates of latitude and longitude were attributed. MCMC analyses were performed in BEAST v1.10.4, running in duplicate for 50 million interactions and sampling every 10,000 steps in the chain. Convergence for each run was assessed in Tracer (effective sample size for all relevant model parameters >200). MCC trees for each run were summarized using TreeAnnotator after discarding the initial 10% as burn-in. Finally, we used the R package 'seraphim' version 1.0 (*15*) to extract and map spatiotemporal information embedded in the posterior trees.

Epidemiologic Data

Epidemiologic data of weekly fatal, notified and laboratory confirmed cases CHIKV in Paraguay from 2013 to 2023 (Figure 1) were obtained and curated from the PAHO data repository for Chikungunya (*16*). Confirmed infections are defined as a suspected or probable chikungunya case with a chikungunya test with positive result (as stated on the PAHO platform).

Epidemiologic data (Appendix 1 Figure 1) was provided by Dirección General de Vigilancia de la Salud del Ministerio de Paraguay (DGVS), including suspected, probable and confirmed CHIKV infections between 2015 and 2023 (*17*). Suspected infections are defined as any person with sudden onset of fever and arthralgia or disabling arthritis of sudden onset not explained by another medical condition. Probable infections are defined as any suspected case

with a positive laboratory result for CHIKV (IgM ELISA) or any suspected case of CHIKV with an epidemiologic link with a confirmed case. Confirmed infections are any suspected or probable case of CHIKV that includes real-time RT-PCR or viral isolation. When epidemiologic data are presented, we aggregate suspected and probable infections into a single, suspected category.

Sample Metadata

Samples were selected for sequencing based on a Ct value (≤35) and availability of epidemiologic metadata, such as date of symptom onset, date of sample collection, sex, age, municipality of residence, symptoms, and disease classification (Appendix 2). Patients were classified based on their clinical outcomes: Outpatient, Inpatient, intensive care unit (ICU), and fatal cases.

Temperature Data

Monthly temperature data for Paraguay was extracted from Copernicus.eu satellite climate data (*18*). We summarized the temperature data by calculating the minimum, mean and maximum per year.

Generalized Additive Model of Sample Sequence Coverage versus Ct

We consider the sequencing coverage of each sample (between 0 and 1) as a probability that all genome sites are sequenced with success. For this, we augmented the dataset by counting the number of successful and unsuccessful events (sequencing of sites) per sample, from which we model a binomial based Generalized Additive Model (GAM). GAM was implemented using R v3.6.3 and the package mgcv v1.38.1 (*19*,*20*). We included random effects for the clinical/infection outcome associated with each sample (outcomes) and for each sample independently (ID). The following code snippet summarizes this approach:

```
studyData_success<- data
studyData_success$N<- round(genome_size*studyData_success$Coverage)
studyData_failures<- data
studyData_failures$N<- genome_size-round(genome_size*studyData_failures$Coverage)
studyData <- bind_rows(studyData_success %>% mutate(posTest = 1), studyData_failures %>% mutate(posTest = 0))

gam2 <- mgcv::gam(posTest ~ s(CT, bs = "cr") + s(ID, bs = "re") + s(outcomes, bs = "re"),
                  data = studyData, family = binomial(),
                  method = "REML", select=FALSE, weights = studyData$N)
summary(gam2)
```

References

1. Lanciotti RS, Kosoy OL, Laven JJ, Velez JO, Lambert AJ, Johnson AJ, et al. Genetic and serologic properties of Zika virus associated with an epidemic, Yap State, Micronesia, 2007. Emerg Infect Dis. 2008;14:1232–9. PubMed https://doi.org/10.3201/eid1408.080287

2. Santiago GA, Vergne E, Quiles Y, Cosme J, Vazquez J, Medina JF, et al. Analytical and clinical performance of the CDC real time RT-PCR assay for detection and typing of dengue virus. PLoS Negl Trop Dis. 2013;7:e2311. PubMed https://doi.org/10.1371/journal.pntd.0002311

3. Lanciotti RS, Kosoy OL, Laven JJ, Panella AJ, Velez JO, Lambert AJ, et al. Chikungunya virus in US travelers returning from India, 2006. Emerg Infect Dis. 2007;13:764–7. PubMed https://doi.org/10.3201/eid1305.070015

4. Quick J, Grubaugh ND, Pullan ST, Claro IM, Smith AD, Gangavarapu K, et al. Multiplex PCR method for MinION and Illumina sequencing of Zika and other virus genomes directly from clinical samples. Nat Protoc. 2017;12:1261–76. PubMed https://doi.org/10.1038/nprot.2017.066

5. Vilsker M, Moosa Y, Nooij S, Fonseca V, Ghysens Y, Dumon K, et al. Genome Detective: an automated system for virus identification from high-throughput sequencing data. Bioinformatics. 2019;35:871–3. PubMed https://doi.org/10.1093/bioinformatics/bty695

6. Katoh K, Rozewicki J, Yamada KD. MAFFT online service: multiple sequence alignment, interactive sequence choice and visualization. Brief Bioinform. 2019;20:1160–6. PubMed https://doi.org/10.1093/bib/bbx108

7. Larsson A. AliView: a fast and lightweight alignment viewer and editor for large datasets. Bioinformatics. 2014;30:3276–8. PubMed https://doi.org/10.1093/bioinformatics/btu531

8. Nguyen L-T, Schmidt HA, von Haeseler A, Minh BQ. IQ-TREE: a fast and effective stochastic algorithm for estimating maximum-likelihood phylogenies. Mol Biol Evol. 2015;32:268–74. PubMed https://doi.org/10.1093/molbev/msu300

9. Rambaut A, Lam TT, Max Carvalho L, Pybus OG. Exploring the temporal structure of heterochronous sequences using TempEst (formerly Path-O-Gen). Virus Evol. 2016;2:vew007. PubMed https://doi.org/10.1093/ve/vew007

10. Suchard MA, Lemey P, Baele G, Ayres DL, Drummond AJ, Rambaut A. Bayesian phylogenetic and phylodynamic data integration using BEAST 1.10. Virus Evol. 2018;4:vey016. PubMed https://doi.org/10.1093/ve/vey016

11. Baele G, Li WL, Drummond AJ, Suchard MA, Lemey P. Accurate model selection of relaxed molecular clocks in bayesian phylogenetics. Mol Biol Evol. 2013;30:239–43. PubMed https://doi.org/10.1093/molbev/mss243

12. Lemey P, Rambaut A, Welch JJ, Suchard MA. Phylogeography takes a relaxed random walk in continuous space and time. Mol Biol Evol. 2010;27:1877–85. PubMed https://doi.org/10.1093/molbev/msq067

13. Pybus OG, Suchard MA, Lemey P, Bernardin FJ, Rambaut A, Crawford FW, et al. Unifying the spatial epidemiology and molecular evolution of emerging epidemics. Proc Natl Acad Sci U S A. 2012;109:15066–71. PubMed https://doi.org/10.1073/pnas.1206598109

14. Dellicour S, Gill MS, Faria NR, Rambaut A, Pybus OG, Suchard MA, et al. Relax, keep walking: a practical guide to continuous phylogeographic inference with BEAST. Mol Biol Evol. 2021;38:3486–93. PubMed https://doi.org/10.1093/molbev/msab031

15. Dellicour S, Rose R, Faria NR, Lemey P, Pybus OG. SERAPHIM: studying environmental rasters and phylogenetically informed movements. Bioinformatics. 2016;32:3204–6. PubMed https://doi.org/10.1093/bioinformatics/btw384

16. Pan-American Health Organization. CHIKV Weekly Report. PAHO. 2023 [cited 2023 Jul 15]. https://www3.paho.org/data/index.php/en/mnu-topics/chikv-en/550-chikv-weekly-en.html

17. Paraguayan Ministry of Health. DGVS, Dirección General de Vigilancia de la Salud del Paraguay, 2023 [cited 2023 Jul 15]. https://dgvs.mspbs.gov.py/enfermedades/chikungunya/

18. Copernicus Climate Data Store. Essential climate variables for assessment of climate variability from 1979 to present [cited 2023 Jul 15]. https://cds.climate.copernicus.eu/cdsapp#!/dataset/ecv-for-climate-change

19. Pedersen EJ, Miller DL, Simpson GL, Ross N. Hierarchical generalized additive models in ecology: an introduction with mgcv. PeerJ. 2019;7:e6876. PubMed https://doi.org/10.7717/peerj.6876

20. The R Project for Statistical Computing [cited 4 Apr 2023]. https://www.R-project.org/

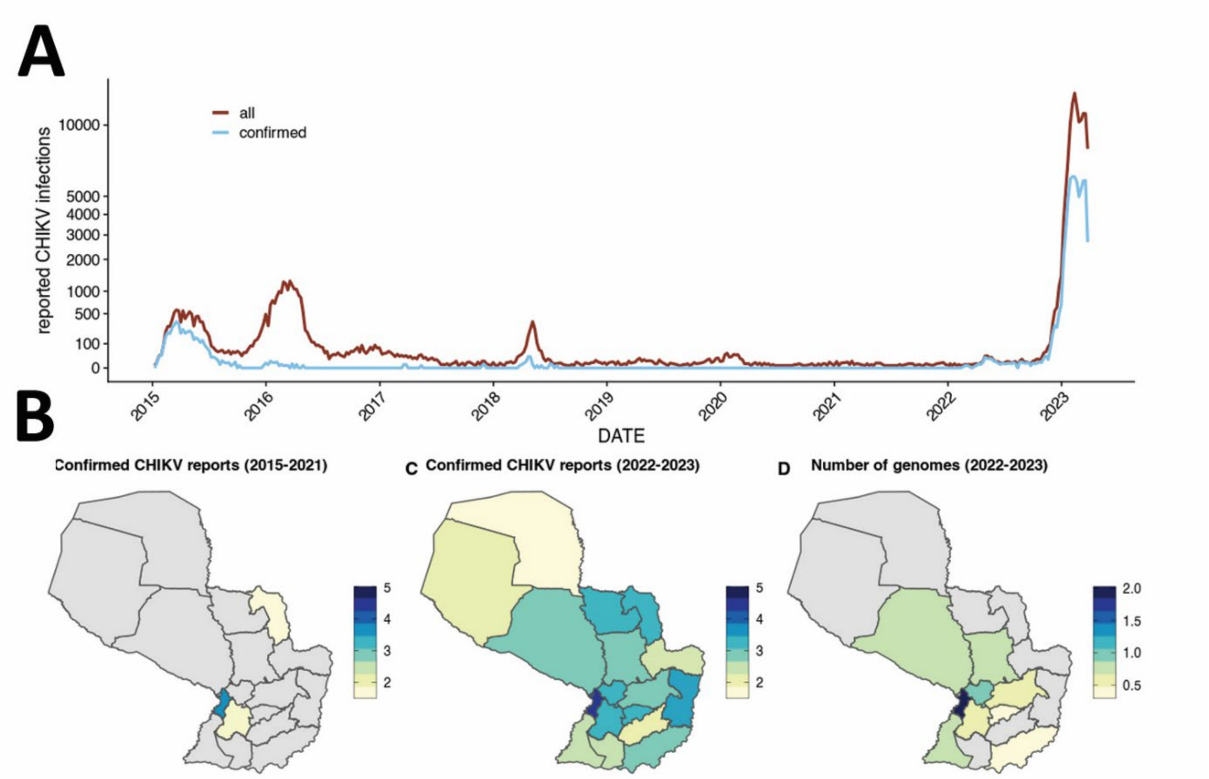

Appendix 1 Figure 1. CHIKV reported infections and sequencing effort. A) Confirmed (in blue) and suspected plus confirmed (in red) CHIKV infections aggregated at the country level (Paraguay) as reported by Dirección General de Vigilancia de la Salud del Ministerio de Paraguay between 2015 and March 2023. B) Spatial distribution of the sum of confirmed CHIKV infections per district in Paraguay between 2015 and 2021.

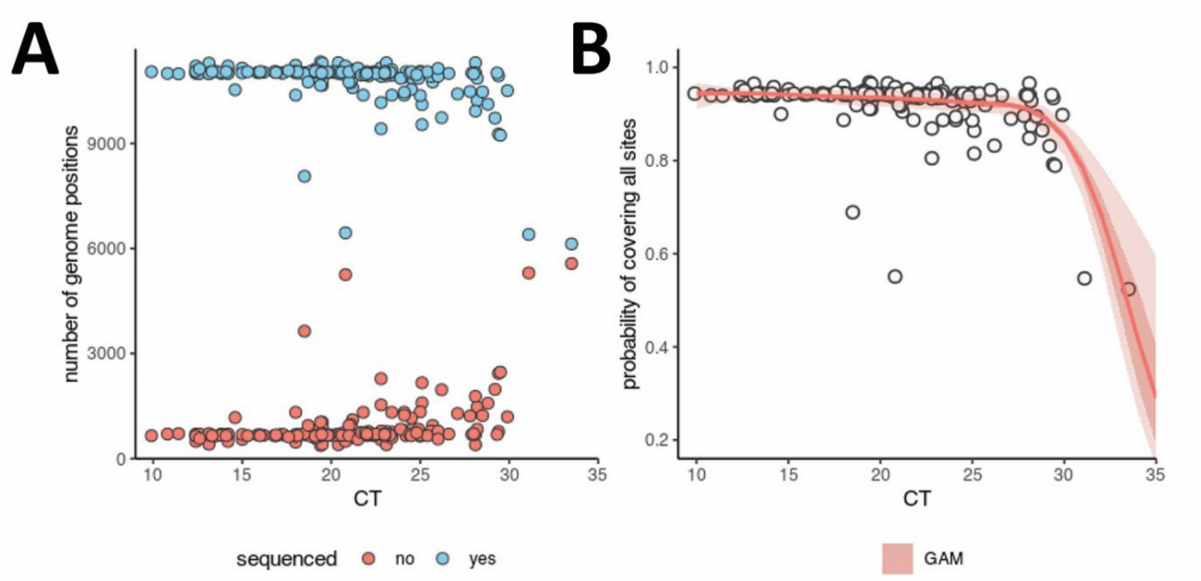

Appendix 1 Figure 2. Generalized additive model of sample sequence coverage versus Ct. A) Augmented fitted data as detailed in the supplementary text. B) GAM predicted the probability of covering all genome sites with sequencing depending on the Ct of each sample. Points are the samples, red line indicates mean predicted probability, dark shaded area indicates 50% CI, and light shaded area indicates 95% CI. Ct, cycle threshold; GAM, genome architecture mapping.

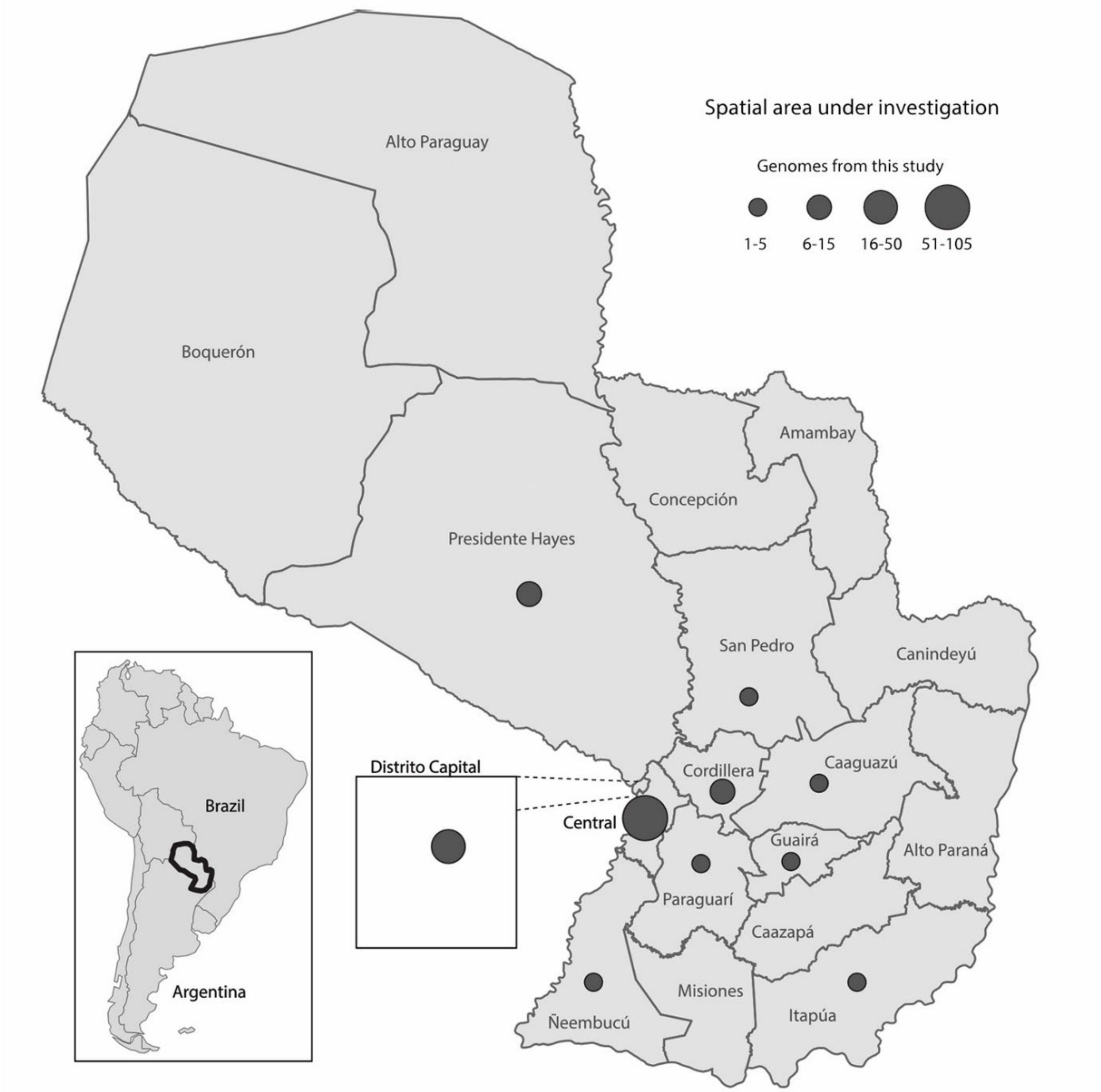

Appendix 1 Figure 3. Spatiotemporal reconstruction of the spread of CHIKV ECSA in Paraguay. Circles represent nodes of the maximum clade credibility phylogeny, colored according to their inferred time of occurrence (scale shown). Shaded areas represent 80% highest posterior density interval and depict uncertainty of the phylogeographic estimates for each node. Solid curved lines indicate links between nodes and directionality of movement. Differences in population density are shown on a gray-white scale..

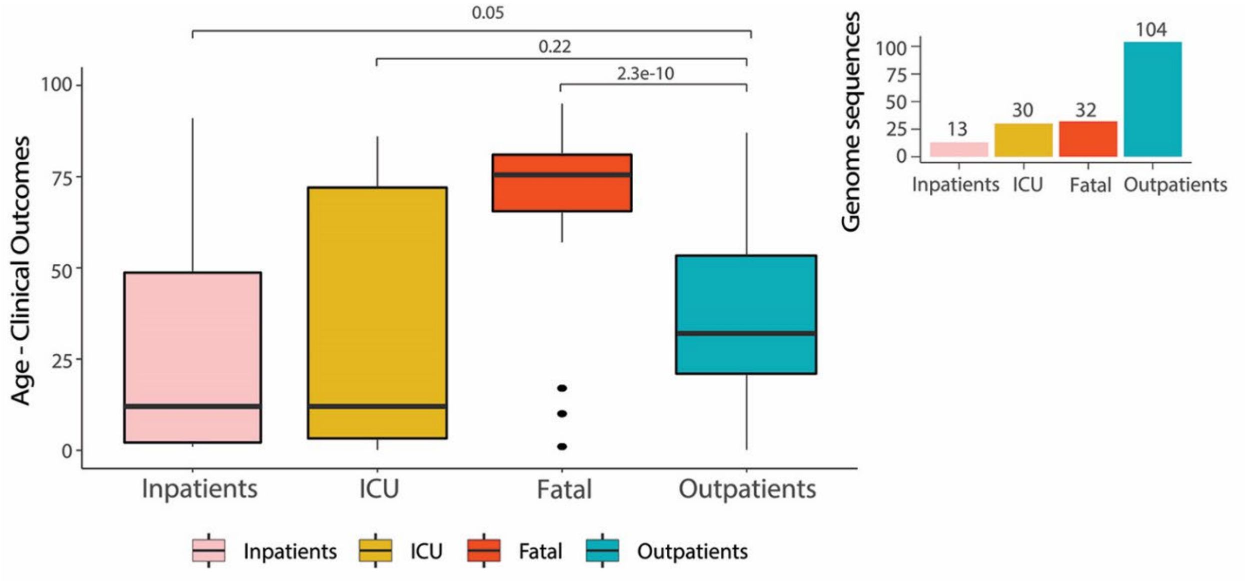

Appendix 1 Figure 4. Boxplot of the patient's (representing the study population) age and clinical outcomes value distribution. The Kruskal-Wallis non-parametric approach was used to determine the strength of association within the different clinical outcomes.

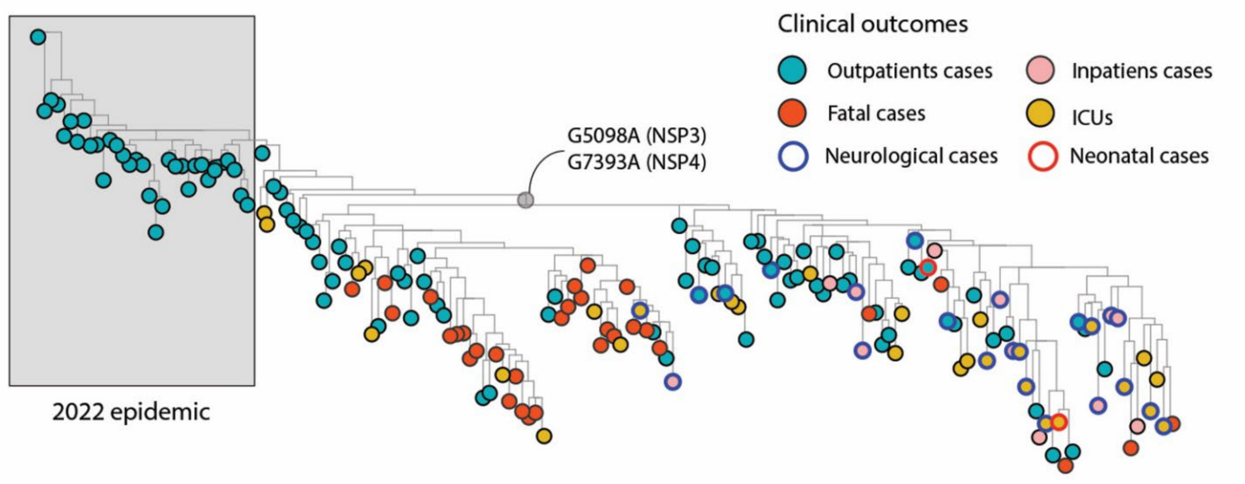

Appendix 1 Figure 5. Spatiotemporal reconstruction of the spread of CHIKV ECSA in Paraguay. Circles represent nodes of the maximum clade credibility phylogeny, colored according to their inferred time of occurrence (scale shown). Shaded areas represent 80% highest posterior density interval and depict uncertainty of the phylogeographic estimates for each node. Solid curved lines indicate links between nodes and directionality of movement. Differences in population density are shown on a gray-white scale. ICU, intensive care unit.
